# Beyond the Binding Site: In Vivo Identification of *tbx2, smarca5* and *wnt5b* as Molecular Targets of CNBP during Embryonic Development

**DOI:** 10.1371/journal.pone.0063234

**Published:** 2013-05-07

**Authors:** Pablo Armas, Ezequiel Margarit, Valeria S. Mouguelar, Miguel L. Allende, Nora B. Calcaterra

**Affiliations:** 1 Instituto de Biología Molecular y Celular de Rosario (IBR), Consejo Nacional de Investigaciones Científicas y Técnicas (CONICET) - Facultad de Ciencias Bioquímicas y Farmacéuticas, Universidad Nacional de Rosario (UNR), Ocampo y Esmeralda, (S2000FHQ) Rosario, Argentina; 2 FONDAP Center for Genome Regulation, Facultad de Ciencias, Universidad de Chile, Santiago, Chile; Karlsruhe Institute of Technology, Germany

## Abstract

CNBP is a nucleic acid chaperone implicated in vertebrate craniofacial development, as well as in myotonic dystrophy type 2 (DM2) and sporadic inclusion body myositis (sIBM) human muscle diseases. CNBP is highly conserved among vertebrates and has been implicated in transcriptional regulation; however, its DNA binding sites and molecular targets remain elusive. The main goal of this work was to identify CNBP DNA binding sites that might reveal target genes involved in vertebrate embryonic development. To accomplish this, we used a recently described yeast one-hybrid assay to identify DNA sequences bound *in vivo* by CNBP. Bioinformatic analyses revealed that these sequences are G-enriched and show high frequency of putative G-quadruplex DNA secondary structure. Moreover, an *in silico* approach enabled us to establish the CNBP DNA-binding site and to predict CNBP putative targets based on gene ontology terms and synexpression with CNBP. The direct interaction between CNBP and candidate genes was proved by EMSA and ChIP assays. Besides, the role of CNBP upon the identified genes was validated in loss-of-function experiments in developing zebrafish. We successfully confirmed that CNBP up-regulates *tbx2b* and *smarca5,* and down-regulates *wnt5b* gene expression. The highly stringent strategy used in this work allowed us to identify new CNBP target genes functionally important in different contexts of vertebrate embryonic development. Furthermore, it represents a novel approach toward understanding the biological function and regulatory networks involving CNBP in the biology of vertebrates.

## Introduction

CNBP, formerly ZNF9, is a highly conserved nucleic acid chaperone [1] involved in proper organization of the zebrafish, chick, and mouse forebrain [2]–[5]. CNBP loss-of-function adversely affects the formation and survival of a subpopulation of cranial neural crest (CNC) cells, leading to a reduction in size and even loss of selected pharyngeal and craniofacial cartilaginous structures in the developing zebrafish [4], [5]. CNBP is also involved in muscle human diseases, such as myotonic dystrophy type 2 (DM2, proximal myotonic myopathy, OMIM # 602668) [6] and the age-related sporadic inclusion body myositis (sIBM) [7]. DM2 is an autosomal dominant multisystemic disease caused by an expansion of intronic CCTG repeats in intron 1 of the human *CNBP* gene, which leads to a disruption of RNA metabolism in patients’ tissues by accumulation of untranslated CCUGn RNAs [8]. Furthermore, lower levels of CNBP in DM2 muscle cells lead to a reduction of proteins of the translational apparatus, which results in an overall reduction of global protein synthesis [9], [10]. sIBM is an inflammatory muscle disease characterized by abnormal accumulation of intra-muscle fiber aggregates composed mainly of amyloid precursor protein (APP) and β-amyloid peptide (Aβ) [11]. APP is commonly overexpressed in the disease state [12], and its overexpression strikingly reduces CNBP expression [7]. The molecular mechanisms responsible for this reciprocal regulation have not been completely elucidated yet.

CNBP binds to single-stranded DNA (ssDNA) and RNA and participates in the control of translational and transcriptional processes [1]. Data gathered so far strongly suggests that CNBP is involved in controlling cell death and proliferation rates through gene expression regulation [1]. However, how CNBP fulfills its biological function is still largely unknown likely because of the scant information available on the molecular targets of CNBP.

In this work, we applied a recently described yeast one-hybrid assay [13] with the aim of identifying CNBP DNA-binding sites and to search for CNBP gene target candidates using mouse and zebrafish genomic libraries. Various bioinformatic analyses enabled us to find the CNBP eukaryotic DNA-consensus binding site and to identify a set of genes previously not functionally related to CNBP. Electrophoretic mobility shift assays (EMSA) and chromatin immunoprecipitation (ChIP) revealed a direct interaction between CNBP and the consensus-binding site found *in silico*, as well as candidate genes. Moreover, the role of CNBP on the expression of candidate targets was further assessed in developing zebrafish depleted of CNBP by microinjecting a specific morpholino, validated in a previous report [5]. This work represents a novel approach towards understanding the biological function and regulatory networks involving CNBP in the biology of vertebrates.

## Results

### Whole-genome Screening for CNBP Target Genes

In order to identify CNBP DNA targets in the zebrafish and mouse genomes, screens were performed using a recently described assay based on libraries containing random genomic fragments upstream of the yeast *URA3* gene in yeast cells [13]. Briefly, *MATa* haploid yeast strains containing mouse and zebrafish genome libraries were separately mated to haploid *MATα* yeast strain expressing zebrafish CNBP fused to the GAL4 activation domain. Mating efficiency was ≈8% for the zebrafish library and ≈17% for the mouse library. We obtained 207 Ura+ diploid clones from the mouse library mating and 85 from the zebrafish library mating. Genomic fragments were PCR-amplified directly from yeast colonies or from purified plasmids and then sequenced using nested primers.

### Computational Analysis of the Identified Genomic Sequences

#### Mouse genomic sequences

Of the 207 clones rescued from mouse library, DNA sequences were obtained for 193 genomic fragments (14 could not be sequenced even after several attempts using both pairs of primers and purified plasmids; Table 1). Clones were analyzed to identify and remove vector sequences or those not belonging to *Mus musculus,* and subsequently mapped to the mouse genome. The 193 sequences were further reduced to 85 unique sequences due to multiple representation of some clones, and 118 subsequences (Table 1 and Table S1) due to chimeric clones present in the library [13]. The 85 unique sequences comprised 35,880 base pairs, and showed a GC content of 44.8±4.0% and an average length of 422±204 bp (Table 1 and Table S1). Regarding the 118 sub-sequences, 44.1% mapped inside transcriptional units, 2.5% into promoter regions (defined as regions spanning <1 kbp from reported transcriptional start sites), 31.4% in intergenic regions spanning >10 kbp up- and down- stream from reported transcriptional units, and 17.8% in intergenic regions spanning <10 kbp up- and down- stream from reported transcriptional units (Table 1 and Table S1). Finally, 4.2% of subsequences could not be mapped since they showed homology to numerous loci in the mouse genome (indicated as multiple sites in Table S1).

**Table 1:** Screening results and data analysis for *Mus musculus* and *Danio rerio* libraries.

Library screened	Ura+ colonies	Sequencedclones	Unique Sequences	Total bases	GC content (%)	Average seq.Size (bp)	Sub-sequences	Subsequences		
								Localization	n	%
***Mus musculus***	207	193	85	35,880	44.8±4.0	422±204	118	Transcriptional unit	52	44.1
								Promoter (<−1 kbp)	3	2.5
								Intergenic (<10 kbp)	21	17.8
								Intergenic (>10 kbp)	37	31.4
								Unmapped	5	4.2
***Danio rerio***	85	76	11	4,229	43.3±5.1	384±242	19	Transcriptional unit	11	57.9
								Promoter (<−1 kbp)	–	–
								Intergenic (<10 kbp)	2	10.5
								Intergenic (>10 kbp)	5	26.4
								Unmapped	1	5.3

#### Zebrafish genomic sequences

A similar computational analysis was performed on the 85 clones obtained from the zebrafish library. In this case, 76 clones could be sequenced and 9 failed to result in readable sequence. The 76 sequences produced 11 unique and 19 subsequences (Table 1 and Table S1). Sequences corresponded to 4,229 base pairs, and presented a GC content of 43.3±5.1% and length of 384±242 bp (Table 1 and Table S1). Regarding the 19 subsequences, 57.9% were located within transcriptional units, 26.4% in intergenic regions spanning >10 kbp up- and down- stream from reported transcriptional units and 10.5% in intergenic regions spanning <10 kbp up- and down-stream from reported transcriptional units. No sequences were found in proximal promoter regions (Table 1). Again, due to homology with multiple sites, 5.3% of the subsequences could not be mapped to a single chromosomal position (Table S1).

#### Determination of CNBP consensus DNA-binding site

**Figure 1:**
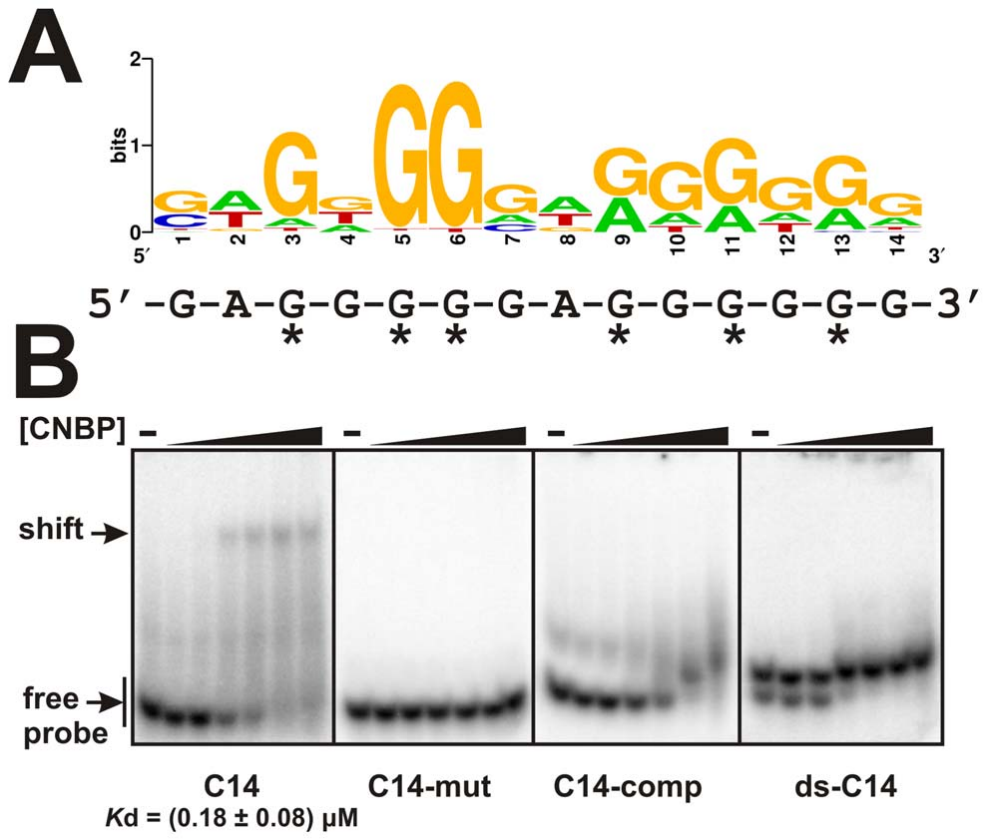
Consensus sequence for CNBP DNA-binding site. (**A**) A consensus CNBP binding sequence was predicted by applying the MEME program to the 85 and 11 unique sequences obtained from *M. musculus* and *D. rerio* libraries, respectively. The logo shown was generated using Weblogo. The 14-nucleotide consensus sequence used for probes design is shown below the logo. Asterisks indicate the six more conserved guanine residues. (**B**) EMSAs were performed using labeled ssDNA probes (C14, C14-mut, C14-comp and dsC14) and increasing concentrations of recombinant CNBP (0.015, 0.050, 0.15, 0.50, 1.50 and 5 µM). Free and shifted probes are indicated by arrows at the left of the gels. *K*_d_ for C14 is indicated below the gel.

The 96 unique sequences (85 from mouse and 11 from zebrafish libraries) were analyzed by several algorithms using Tmod [14]. Different consensus lengths were tried resulting in a similar 14-nucleotide motif comprised by a duplication of a central core of five guanosines flanked by an adenine residue (*p*-value 1 e^−40^; Figure 1A and, Tables S1 and S2).

EMSA were performed to confirm CNBP binding to the consensus-binding site found *in silico*. We used a recombinant zebrafish CNBP and a set of probes shown in Table S3. CNBP bound to the 14-nucleotide G-rich consensus sequence (C14) with high affinity but failed to bind to a mutant probe (C14-mut) in which the six more conserved guanines were replaced by adenine residues (Figure 1B). Moreover, CNBP failed to bind to the complementary C-rich sequence (C14-comp) and to a double-stranded probe (ds-C14) formed by C14 and C14-comp (Figure 1B). Data indicate that CNBP has a preference for single-stranded G-enriched sequences, as we previously reported [15].

#### Searching for putative G-quadruplex DNA-forming sequences (PQS)

G-rich sequences that contain stretches of tandem guanines can form intramolecular four-stranded structures called G-quadruplexes or G4s. CNBP was previously described to modulate the formation of a G4 structure within the *c-Myc* promoter [16]. This finding led us to search for PQS in zebrafish and mouse sequences recovered in our screen. PQS were searched *in silico* using Quadparser software setting the parameters to find out 2 (G2) or 3 (G3) stacked G-quartets with loop lengths between 1 to 15 nucleotides. Among the 19 *Danio rerio* subsequences, 14 contained G2 and one contained G3 PQS (Table 2 and Table S1). In *Mus musculus*, G2 and G3 PQS were found in 74 and 27 subsequences, respectively (Table 2 and Table S1). Most of PQS were located within transcriptional units, mainly in intronic sequences (Table 2 and Table S1).

**Table 2:** Quadruplex screening in *M. musculus* and *D. rerio* mapped sequences.

	Putative Quadruplex sequences (a)				PQS density (b)	
	Localization	G2	G3	ND	Library rescued sequences	Genome
***Mus musculus***	Transcriptional Unit	35 (29.7%)	8 (6.8%)	9 (7.6%)	10.03	6.52±1.39
	Promoter (<−1 kbp)	3 (2.5%)	–	–		
	Intergenic (<10 kbp)	12 (10.2%)	8 (6.8%)	1 (0.8%)		
	Intergenic (>10 kbp)	21 (17.8%)	11 (9.3%)	5 (4.2%)		
	Unmapped	3 (2.5%)	–	2 (1.7%)		
***Danio rerio***	Transcriptional Unit	8 (42.1%)	1 (5.3%)	2 (10.5%)	6.15	3.44±0.11
	Promoter (<−1 kbp)	–	–	–		
	Intergenic (<10 kbp)	2 (10.5%)	–	–		
	Intergenic (>10 kbp)	4 (21.1%)	–	1 (5.3%)		
	Unmapped	–	–	1 (5.3%)		

**(a)** Values show the number of subsequences containing at least one G2 or G3 quadruplex with loop lengths of 1 to 15 nucleotides. Percentages reflect the number of subsequences containing G3, G2 or not containing quadruplexes (ND) relative to the total number of sequences studied.

**(b)** PQS density calculated considering the number of G2 quadruplexes with loop lengths of 1 to 15 nucleotides per kbp studied.

**PQS**, Putative Quadruplex Sequences.

The PQS frequency reflects an enrichment of PQS in CNBP bound sequences when compared to the PQS frequencies obtained for the *M. musculus* or *D. rerio* genomes (Table 2). This high PQS frequency reinforces the notion of CNBP as a transcriptional regulator through G4-folding modulation.

Collectively, our screen carried out in yeast enabled us to identify sequences bound *in vivo* by CNBP, which were enriched in GC, and showed the G(A/T)G_5_(A/T)G_6_ consensus and high PQS frequencies. These findings led us to carry out a more accurate identification of CNBP molecular targets, as described below.

### *In silico* Analysis and Selection of Putative CNBP Target Genes

**Figure 2:**
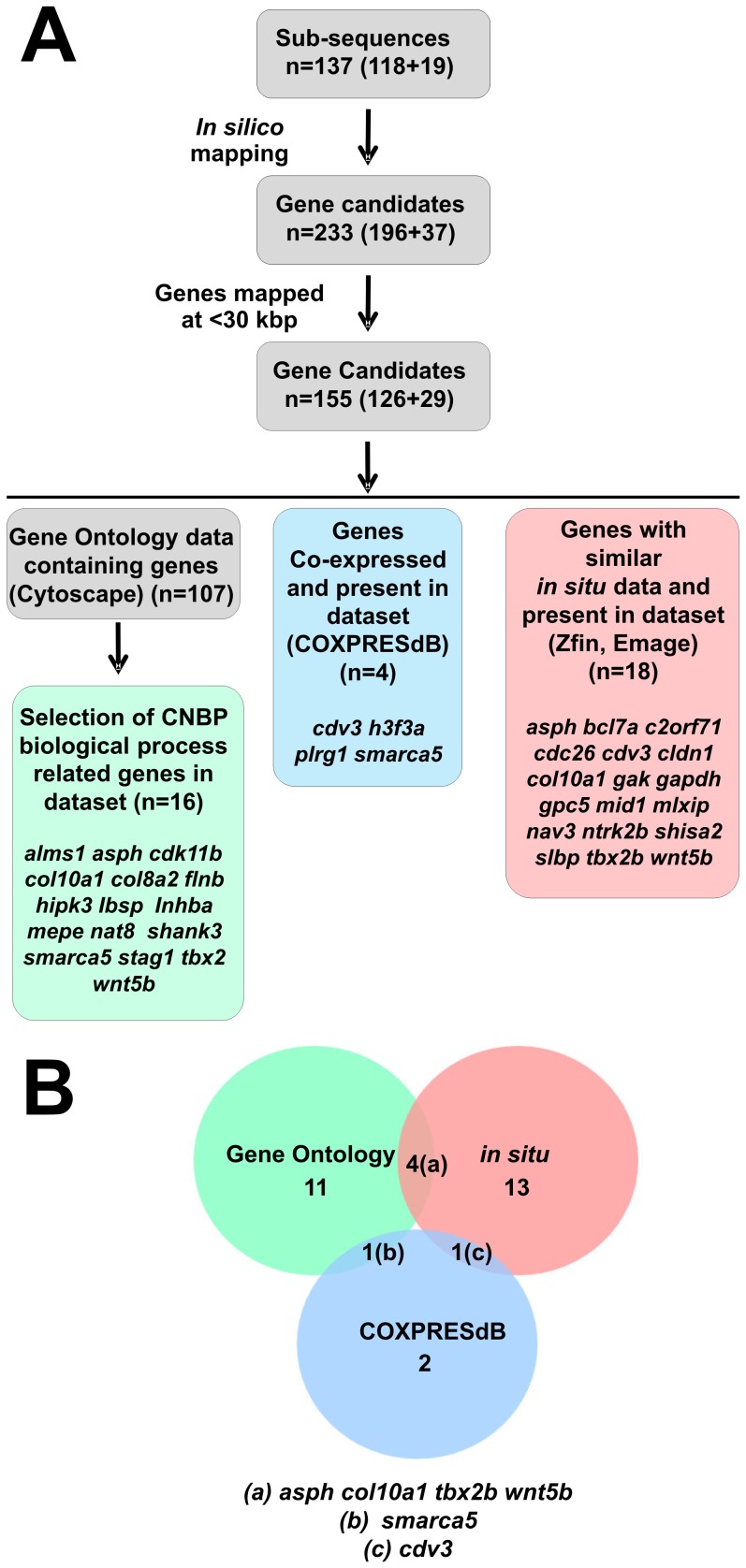
Selection strategy of putative CNBP target genes by *in silico* analysis. (**A**) The 137 sub-sequences (118 from the mouse library +19 from the zebrafish library) found in yeast screen were then used for *in silico* mapping of putative CNBP target genes. The first selection strategy was to choose from genes proximal to sequences bound by CNBP (233 genes = 196 from mouse +37 from zebrafish) those mapping at <30 kbp (155 genes = 126 from mouse +29 from zebrafish). These 155 genes were analyzed by three independent criteria: (i) using the Biological Networks Gene Ontology (BiNGO) tool to select 107 genes with Gene Ontology (GO) terms and then selecting genes classified in GO terms related to CNBP such as cell proliferation, cranial morphogenesis and muscle system processes (16 genes = 14 from mouse +2 from zebrafish); (ii) analyzing co-expression with *cnbp* using mouse and zebrafish microarray databases (COXPRESdB) (4 genes from mouse); and (iii) analyzing synexpression with cnbp using mouse and zebrafish expression databases (Emage, Zfin, respectively) (18 genes = 3 from mouse +15 from zebrafish). (**B**) The three data sets were intersected using BioVenn software. Four genes were retrieved in GO and ISH intersection (*tbx2*, *wnt5b*, *asph* and *col10a1*) and one gene in GO and microarray intersection (*smarca5*). Intersection of microarrays and ISH data sets rendered one gene (*cdv3*) and no genes were obtained in the intersection of the three data sets.

The sub-sequences found in yeast screen were subsequently used for *in silico* mapping of putative CNBP target genes. Analyses showed that the identified subsequences mapped inside or around 233 genes (196 from mouse +37 from zebrafish) (Table S1). To retrieve genes potentially regulated by CNBP, we selected for further study the 155 genes (126 from mouse +29 from zebrafish) that mapped at <30 kbp from the identified subsequences (Figure 2A and Table S4).

Genes were analyzed by the Biological Networks Gene Ontology (BiNGO) tool [17] to find those with known biological function. A hundred and seven genes could be linked to particular Gene Ontology (GO) terms (Figure 2A). There was no apparent enrichment in any GO category (Table S4). Therefore, we manually curated them based on the biological roles reported for CNBP [1]. A list of 16 candidate genes was retrieved by selecting genes classified in GO terms including cell proliferation, cranial morphogenesis and muscle system processes (Figure 2A and Table S4).

On the other hand, the 155 genes were collated with genes co-expressed with *cnbp* in mouse and zebrafish microarray (Table S5) and in *in situ* hybridization (ISH) databases (Table S6). Four genes were found to be co-expressed with CNBP in microarrays (all four derived from the *Mus musculus* data set; Figure 2A) and 18 genes using ISH databases (15 from *Danio rerio* and 3 from *Mus musculus* data sets; Figure 2A). Finally, the GO and synexpressed gene sets were intersected using BioVenn [18]. The GO and ISH intersection retrieved four genes (*tbx2, wnt5b, asph* and *col10a1*) while the GO and microarray intersection retrieved one gene (*smarca5*) (Figure 2B). An additional gene, *cdv3*, was found in the intersection of microarrays and ISH data sets but was not further studied since it was not related to the reported biological roles of CNBP. *Asph*, *wnt5b*, *smarca5* and *tbx2* were identified from the mouse genome library while *col10a1* was found when using the zebrafish genome library. No gene was found at the intersection of the three data sets.

**Figure 3:**
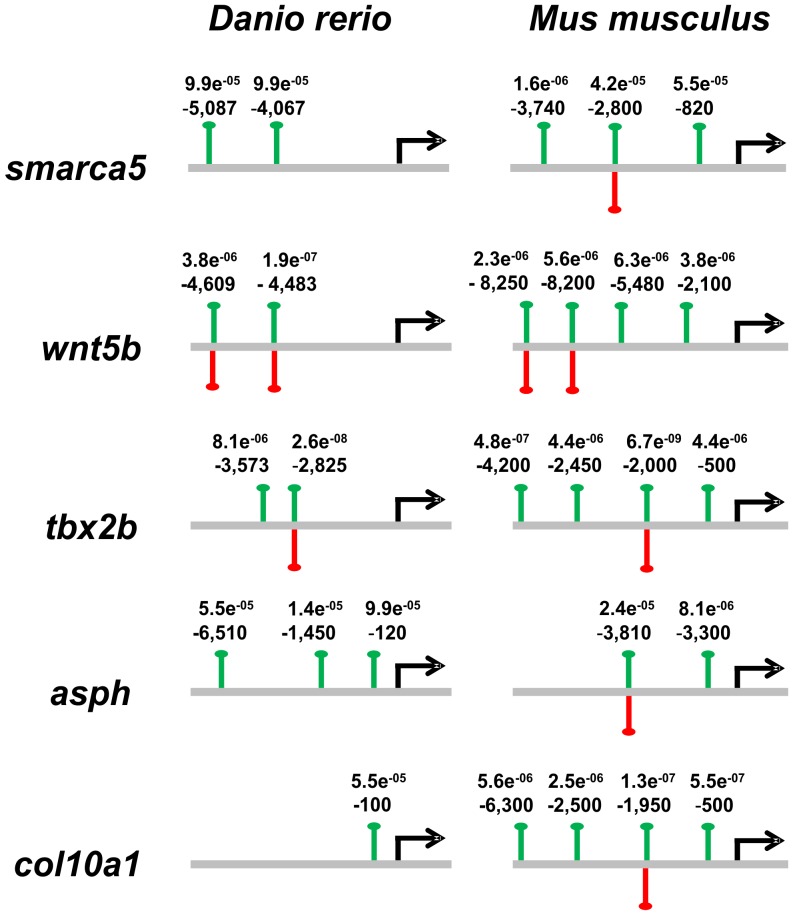
Consensus site and quadruplex mapping in CNBP putative target genes. Upstream regions (<10 kbp) of *asph*, *col10a1*, *smarca5*, *tbx2b* and *wnt5b* genes were scanned, both in zebrafish and in mouse, with the previously determined consensus using MEME/MAST. Green lines show the mapped consensus. Relative positions to the transcription start sites and the p-value of the MEME/MAST prediction are indicated. In addition, G3 putative quadruplex sequences (G3-PQS) were searched for these sequences using Quadparser. Red lines show the G3-PQS positions.

In addition, regions spanning 10 kbp upstream from the transcription start site of the five selected genes were examined for the CNBP consensus-binding site established in this work, as well as for G3-PQS. Several CNBP-binding sites were detected, many of which were found overlapping with G3-PQS (Figure 3).

We are aware that selection criteria for arriving at this list of candidates were highly stringent and may have excluded putative CNBP targets. However, we attempted to arrive at a set of genes that had a high probability of being *bona fide* CNBP targets.

### Validation of Identified Genes as CNBP Targets

Yeast one-hybrid screening followed by bioinformatic analyses retrieved five novel putative CNBP target genes (Figure 2B). However, the physiological relevance of the obtained candidates may not be clear since they were the result of a heterologous cell-based screening. To address this, the behavior of candidate genes was assessed *in vivo* in CNBP-knock down experiments in developing zebrafish embryos. Embryos at the one/two-cell stage were injected with a Morpholino (MO) that specifically blocks *cnbp* pre-mRNA splicing (spl-MO) or with a miss-paired MO (mis-MO) as control, as reported [5]. Embryonic development was allowed to proceed until 24-hours post-fertilization (hpf), and the expression of candidate genes was measured by quantitative real time RT-PCR (qRT-PCR). Studies were performed on 24-hpf staged embryos because (i) at this developmental stage organogenesis has already started [19] and; (ii) phenotypes of CNBP depleted embryos (CNBP morphants) can be easily distinguished in developing zebrafish [4], [5].

**Figure 4:**
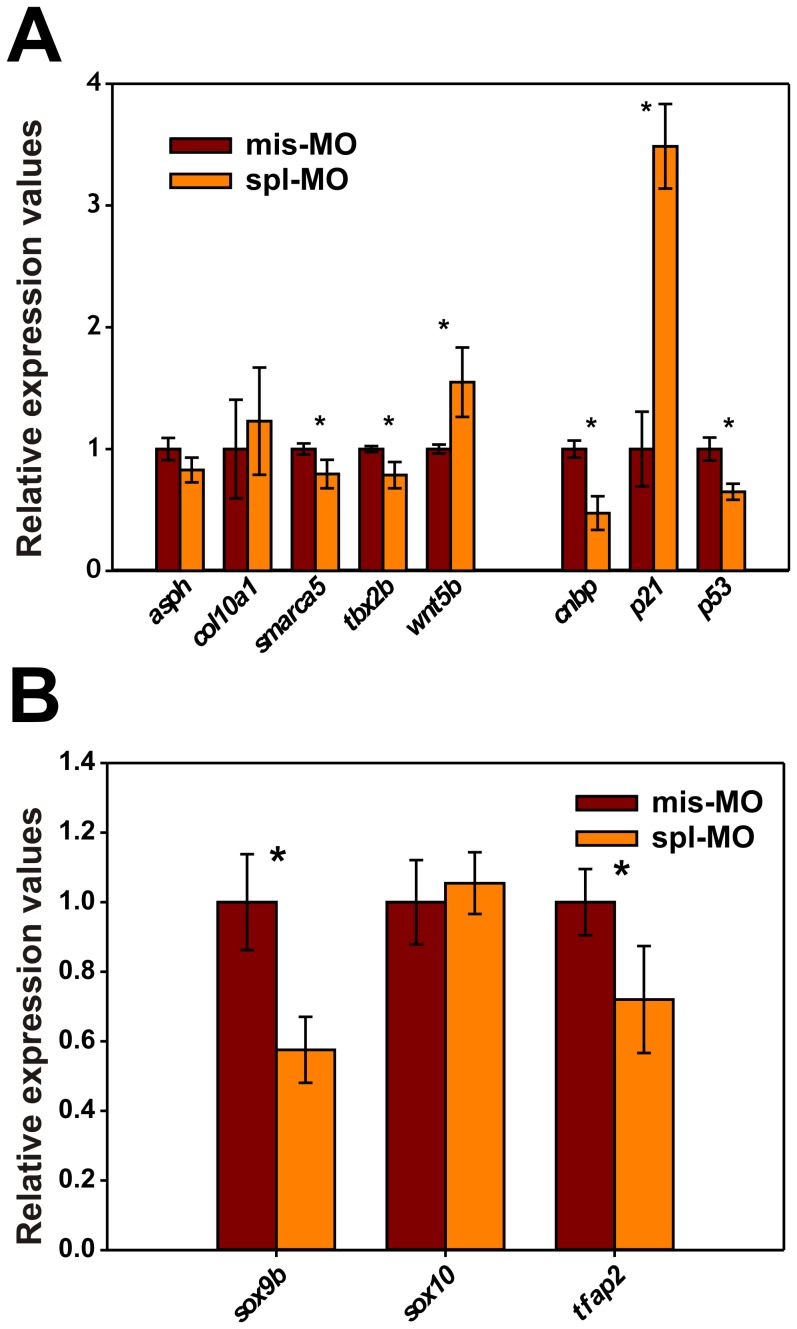
Expression analysis of CNBP target candidates by qRT-PCR. (**A**) Relative gene expression levels of putative CNBP target genes (*asph*, *col10a1*, *smarca5*, *tbx2b* and *wnt5b*), *cnbp* or control genes (*p21* and *p53*) were determined by triplicate using three independent RNA extractions. qRT-PCR was performed on RNA samples obtained from spl-MO or mis-MO morpholinos. Gene expression levels were normalized using *ef1a* and *rpl13a*. (**B**) Relative gene expression levels of *sox9b*, *sox10* and *tfap2* were determined in spl-MO and mis-MO samples. Gene expression levels were normalized using *ef1a* and *rpl13a* and subsequently adjusted to the ‘per embryo total RNA yield’ both in spl-MO or mis-MO samples. In all cases, significant differences (p-values <0.05, n = 3) are labeled with an asterisk.

Spl-MO injection caused a significant reduction of *cnbp* expression levels (47±13%) along with a significant reduction in *smarca5* (20±11%) and *tbx2b* (21±11%) expression. In contrast, we observed a significant increase in *wnt5b* expression levels (55±28%). A regulatory role for CNBP on *asph* and *col10a1* expression could not be validated by qRT-PCR (Figure 4A).

The CNBP loss-of-function effect was previously assessed by microinjecting two different MOs, one of them preventing *cnbp*-mRNA translation (tra-MO) [4] and the other one used in this work (spl-MO) [5]. The specificity of both MOs was further addressed by injecting an mRNA coding for a GFP-CNBP chimera, which rescued morphant phenotypes [4], [5]. However, because MO injection may induce neural cell death by off-target activation of p53 [20], we checked by qRT-PCR the transcriptional levels of *p53* and *p21* in spl-MO injected embryos at 24-hpf. *p53* expression was reduced (34±6%) while *p21* expression was dramatically increased (248±35%) in *cnbp*-morphants (Figure 4A). In addition, we tested the expression levels of the neural crest marker genes *tfap2*, *sox9b* and *sox10* by qRT-PCR. In agreement with previous reports [4], [5], CNBP knock down caused a significant reduction of *tfap2* (72±15%) and *sox9b* (57±9%) expression while *sox10* expression was not affected (Figure 4B).

**Figure 5:**
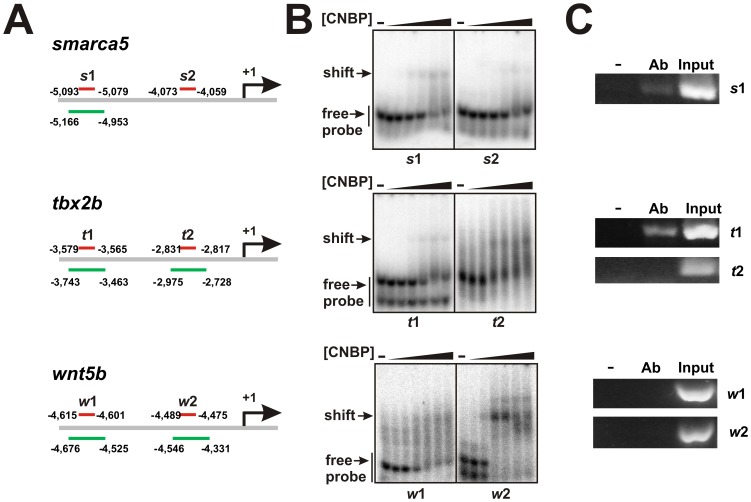
Proving the direct interaction between CNBP and candidate targets. (**A**) Scheme of the upstream regions of zebrafish *smarca5*, *tbx2b* and *wnt5b* genes. Red lines indicate the position of the predicted consensus-like CNBP-binding sites. Green lines indicate genomic regions studied by PCR in ChIP experiments. Numbers indicate positions relative to the transcription start sites (+1). (**B**) EMSAs were performed using labeled ssDNA probes containing the predicted consensus-like CNBP-binding sites from candidate targets and increasing concentrations of recombinant zebrafish CNBP (0.015, 0.05, 0.15, 0.5, 1.5 and 5 µM). Free and shifted probes are indicated by arrows at the left of the gels. (**C**) CNBP-ChIP assays were performed on 24-hpf zebrafish embryos expressing CNBP fused to eGFP. Antibody (Ab), no antibody (-) and input samples were analyzed by PCR for the indicated predicted consensus-like CNBP-binding sites.

The three putative CNBP targets were found from a mouse genome library. However, the CNBP-regulation was assessed in zebrafish developing embryos. Therefore, we performed EMSA (Figure 5) to address whether CNBP binds the consensus-like sequences found in zebrafish orthologous (Figure 3). EMSA were performed using recombinant CNBP and 30-nucleotide probes containing the wild type consensus-like CNBP-binding site found within 10 kbp up-stream *tbx2b*, *smarca5* and *wnt5b* plus eight nucleotides surrounding up-stream and down-stream the consensus-like site. We assessed two putative sites for the regulatory regions of each candidate target gene, *t*1 and *t*2 for *tbx2b*; *w*1 and *w*2 for *wnt5b*; and *s*1 and *s*2 for *smarca5* (Figure 5A). We also assessed their respective mutant versions in the positions of the most conserved guanines (*t*1-mut, *t*2-mut, *w*1-mut, *w*2-mut, *s*1-mut and *s*2-mut). Probes containing the C14, C14-mut, C14-comp or dsC14 sequences plus 8-nucleotide A/T rich flanking sequences (named as C30, C30-mut, C30-comp and ds-C30 respectively) were used as controls. Sequences for all the probes are shown in Table S3. CNBP bound C30 in a similar fashion than C14 (Figure S1A). All wild type probes were bound by CNBP (Figure 5B), while mutations in conserved guanines abrogated the binding to most of them (Figure S1B).

Additionally, we carried out chromatin immunoprecipitation (ChIP) assays to confirm the binding of CNBP and its putative targets *in vivo.* We transiently expressed a zebrafish CNBP-eGFP fusion protein in zebrafish embryos. Fluorescent 24-hpf embryos were selected for testing CNBP transient expression by western blotting (Figure S2). After immunoprecipitation, PCRs were carried out using primers (sequences shown in Table S3) designed to amplify the regions containing the consensus-like CNBP-binding sites identified in each gene (Figure 5A). We could not assess one site for *smarca5 (s*2) due to difficulties in oligonucleotide design. CNBP immunoprecipitated *s*1 and *t*1 but not *t*2, *w*1 and *w*2 (Figure 5C). These results confirm the direct interaction between CNBP and *smarca5* and *tbx2b* control regions. In our experimental conditions, *wnt5b* could not be confirmed *in vivo* as a direct CNBP target. However, ChIP was done using 24-hpf embryos whereas *wnt5b* expression is maxima at 60% epiboly and rather lower at 26-hpf stage [21]. Therefore, the increased *wnt5b* expression detected in by qRT-PCR could reflect an earlier effect of CNBP on this gene during zebrafish embryonic development.

Altogether, EMSA and ChIP results provided validation not only for the direct link between CNBP and *wnt5b*, *tbx2b*, *smarca5* but also for the existence of a high degree of conservation in CNBP targets among vertebrates.

Finally, we further studied CNBP regulation of *tbx2b, smarca5* and *wnt5b* by ISH in CNBP-depleted zebrafish embryos. Besides allowing a relatively rapid verification of gene expression in different CNBP-manipulated embryos, this approach provides spatial information and insights on the nature of CNBP’s biological role.

**Figure 6:**
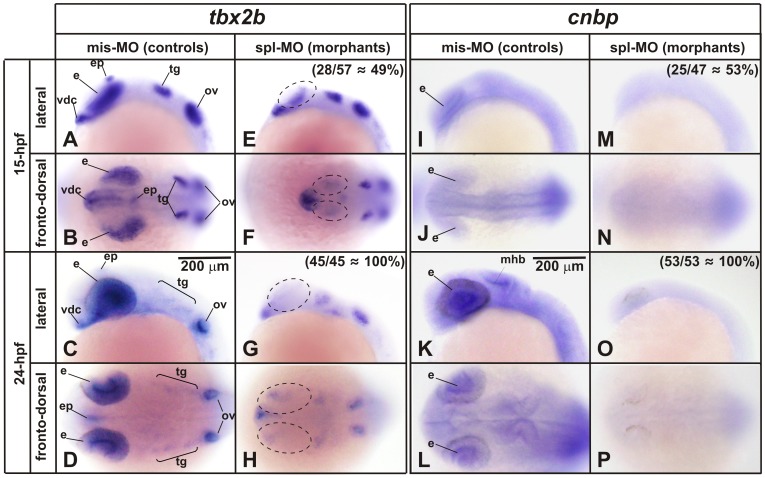
CNBP depletion reduces the expression of *tbx2b* in territories overlapping with *cnbp*. Expression pattern of *tbx2b* and *cnbp* analyzed by whole-mount *in situ* hybridization in 15 and 24-hpf-staged zebrafish embryos microinjected with mis-MO (**A–D and I–L**) or spl-MO (**E-H and M–P**) morpholinos. Anterior is at the left. Scale bar (200 µm) is represented in C and K. *Tbx2b* expression territories affected in morphant embryos are marked with dashed-line ovals. Number and percentages of morphant phenotypes are indicated for each embryo stage. *Cnbp* expression pattern in control embryos (microinjected with mis-MO) is coincident with that described elsewhere [4] and mainly overlapping with *tbx2b* expression in the eye territory. Morphant embryos showed a dramatically reduced *cnbp* expression level in those territories wherein it is normally expressed. vdc, ventral diencephalon; e, eye; ep, epiphysis; mhb, midbrain–hindbrain border; ov, otic vesicle; tg, trigeminal ganglion.

Though qRT-PCR showed that *smarca5* and *wnt5b* expression is modified when decreasing CNBP, no remarkable changes in ISH were detected in the expression pattern of these genes in CNBP morphants (not shown). However, changes in the expression level cannot be ruled out considering that ISH gives qualitative information about expression patterns and may be useful to estimate relative levels of different expression territories, but is limited for comparing global expression levels among different groups of embryos. Moreover, the quantitative sensitivity of ISH is lower than that of qRT-PCR. In contrast, major differences were detected between CNBP morphant and control embryos when analyzing *tbx2b* expression. ISH performed on 15-hpf staged control embryos showed *tbx2b* expression in the ventral diencephalon, eyes (retina), epiphysis, sensory neurons of cranial ganglia (including the trigeminal placode), and otic vesicles (Figure 6A and 6B). In CNBP knock down embryos, the expression of *tbx2b* in the eye territory was markedly affected (Figure 6E and 6F). No significant changes were observed in the expression levels and patterns in the ventral diencephalon, trigeminal placode and otic vesicles. In 24-hpf controls, *tbx2b* expression was detected in the same territories as in 15-hpf with prominent expression in the eyes (mainly the posterior retina), and otic vesicle (Figure 6C and 6D). In 24-hpf morphants, *tbx2b* expression was strongly reduced in the eye (Figure 6G and 6H). Embryos were staged according to morphological parameters; even so, morphants displayed head and eye reductions, as was previously reported [4], [5]. *Tbx2b* (Figure 6A–D) and *cnbp* (Figure 6I–L) expression patterns partially overlap in developing zebrafish embryos. Spl-MO reduced the *cnbp-*mRNA levels in all *cnbp* expression territories (Figure 6M–P) while it mainly affected the *tbx2b* expression in the eye territory (Figure 6E–H), the embryonic territory wherein *cnbp* and *tbx2b* expression is fully overlapping. This fact suggests that *tbx2b* transcription is regulated in a context dependent manner and may depend on CNBP in overlapping expression territories, while it may be controlled by different regulatory factors in other ones.

## Discussion

Even though CNBP was reported as a nucleic acid binding protein involved in several essential cellular processes [1], the full understanding of its biological role in vertebrates is still far from being clear. This is likely due to the scant knowledge gathered so far about the nature of its molecular targets. In this work, we employed a yeast-based one-hybrid genome-wide screen to identify mouse and zebrafish DNA sequences that are bound by CNBP in a cellular context. The same yeast-based method had been successfully applied to identify transcription factor target genes [13], [22]. Identified sequences were analyzed by bioinformatics allowing the definition of a consensus DNA-binding site, as well as a set of putative target genes, whose expression profiles were assessed *in vivo* in a vertebrate model. Among the identified targets, we demonstrated that *tbx2b, smarca5* and *wnt5b* are molecular targets of CNBP during zebrafish embryonic development.

### CNBP Binding Site Features

CNBP binds to single-stranded DNA as well as to RNA molecules; however, it displays a differential behavior according to the nature of the bound nucleic acid [23]. It was recently reported that CNBP binds specifically to G(A/U)(A/U) repeats in RNA molecules [24]. This recognition sequence does not contain PQS motifs. Conversely, we found that DNA sequences bound by CNBP are G-enriched, showing the G(A/T)G_5_(A/T)G_6_ consensus, and contain high PQS frequencies. Therefore, CNBP may not only employ different selectivity for RNA and ssDNA, but also play specific roles in DNA and RNA regulation by means of different biochemical mechanisms.

### Identification of Novel CNBP Gene Targets

The yeast-screen and subsequent bioinformatic analysis allowed the identification of five putative CNBP targets. Three of them, *smarca5, wnt5b* and *tbx2,* showed a genetic relationship with CNBP since their expressions changed in 24-hpf CNBP-depleted zebrafish embryos. It is worth noting that even though *smarca5, wnt5b* and *tbx2* were recovered from the mouse genomic library screen, regulatory regions of their zebrafish orthologous were specifically bound by CNBP. This finding reveals potential conservation of the interaction between CNBP and its putative targets among vertebrates.

A full analysis of the detailed molecular mechanisms responsible for transcriptional regulation by CNBP is beyond the scope of this study. However, the presence of numerous G3-PQS overlapping with CNBP consensus binding sites found in regions spanning 10-kbp upstream from transcriptional start sites of these genes hints at the possibility of transcriptional regulation via G4 folding and/or stabilization.

*Smarca5* encodes the protein Snf2h, the ATPase of ISWI-type chromatin remodeling complexes that were suggested as key regulators of gene transcription during development. Indeed, retrovirus insertional zebrafish mutants for *smarca5* show central nervous system necrosis, decreased head and eye size, and an underdeveloped gut and liver [25]. Of note, this phenotype is partially coincident with that observed in CNBP-depleted zebrafish embryos [4], [5]. Thus, it is tempting to speculate that CNBP depletion may lead to lower *smarca5* transcription, which in turn adversely affects proper embryonic development. Further research is required to address this issue.

*Wnt5b* plays a major role in the regulation of cell shape and movement during zebrafish gastrulation [26]. The *Wnt5b*/*pipetail* mutant phenotype in zebrafish is characterized by defects in tail formation and impaired maturation of the cells that contribute to cartilaginous elements of the head skeleton [27]. Though CNC cells migrate normally to their proper location in *Wnt5b*/*pipetail* mutants, chondrocytes are unable to efficiently undergo the process of intercalation-dependent stacking that allows for proper shaping of elongated cartilage elements [28]. In addition, *Wnt5b* overexpression increases chondroprogenitor cell migration and disrupts the cellular aggregation associated with mesenchymal condensation [29]. Our data indicate that CNBP depletion leads to significant *wnt5b* overexpression, which may partially explain the aberrant cartilage development and impaired mesenchymal condensation observed in CNBP-depleted zebrafish larvae [4], [5].

*Tbx2* functions range from the specification of early embryonic germ layers to the determination of cell fate during organogenesis [30]. In addition, accumulating evidence suggests a role for *Tbx2* in tumorigenesis. Up-regulation of *Tbx2* suppresses the expression of *p14^ARF^* and promotes bypass of senescence either by degradation and inactivation of p53 or by p53-independent pathways [31]. Moreover, down-regulation of *Tbx2* leads to a robust activation of *p21^CIP1^* expression [32]. These data are in complete agreement with those obtained in this study. Indeed, our results showed that CNBP depletion leads to *tbx2* down-regulation and, moreover, to *p21* up-regulation trough a p53-independent pathway. On the other hand, *cnbp* was reported as a gene likely involved in Treacher Collins Syndrome (TCS, OMIM #154500) [33]. TCS is an autosomal dominant craniofacial malformation caused by mutations in the *Treacher Collins-Franceschetti syndrome 1* gene (*TCOF1*, OMIM*606847). In zebrafish, *tcof1* loss-of-function represses *tbx2* expression while up-regulates *ndrg1* (N-myc downregulated gene 1) and *p21* expressions [33]. Since *Tbx2* represses the expression of *ndrg1* acting as a co-repressor through recruitment of EGR1 [34], the role of CNBP as *tbx2* up-regulator may explain not only the increase of *p21* expression detected in *cnbp*- and *tcof1*- morphants but also the increase of *ndrg1* expression detected in *TCS*-like zebrafish embryos. Very little is known about the signaling pathways that regulate the expression of *Tbx2* during early embryonic development. It has been demonstrated that several signaling pathways are closely associated with the expression of *Tbx2*, including transforming growth factor β (TGFβ), fibroblast growth factor (FGF) and Wnt [35], which are key players in cell proliferation, differentiation, and patterning during early embryonic development. This is the first evidence of the developmental regulatory role of CNBP on *tbx2* expression.

**Figure 7:**
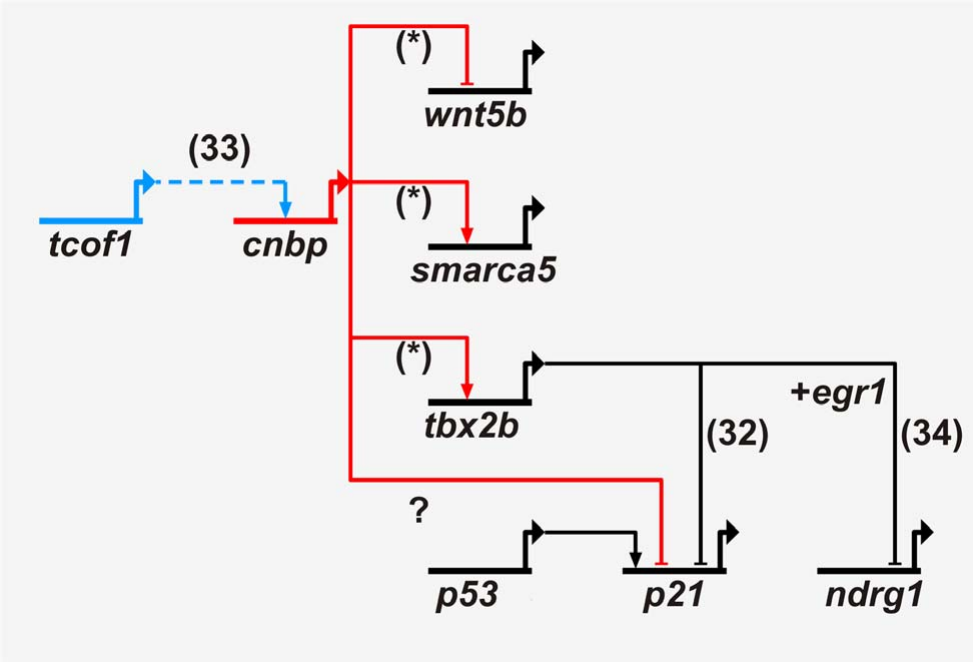
CNBP regulatory genetic network. CNBP regulatory genetic relationships were obtained using Biotapestry software from data achieved in this work (*) as well as previous data from our group [33] and others [32], [34]. Blue lines indicate upstream while red lines indicate downstream CNBP genetic relationships. Black lines indicate other genetic relationships relevant to the CNBP pathway. Arrows indicate positive regulation while break lines indicate negative regulation.

The highly selective strategy used in this work allowed us to identify new CNBP target genes functionally important in vertebrate biology. These data, together with other data from the literature, prompt us to suggest a regulatory network involving CNBP (Figure 7). Further research should be carried out to fully understand the role of CNBP in these pathways.

## Materials and Methods

### Animal Handling and Ethics Statement

This study was carried out in strict accordance with relevant national and international guidelines. Protocols were approved by the Committee on the Ethics of Animal Experiments of the Facultad de Cs. Bioquímicas y Farmacéuticas-UNR, which had been accepted by the Ministerio de Salud de la Nación Argentina (http://www.saludinvestiga.org.ar/comites.asp?num_prov=13). Expedient N° 6060/132; Resolution N° 298/2012.

### Fish and Embryo Rearing

Adult zebrafish were maintained at 28°C on a 14 h light/10 h dark cycle as previously described [36]. Embryos were staged according to development in hours post-fertilization (hpf) at 28°C [19].

### Nucleic Acid Probe and Primer Sequences

All oligonucleotides and primers (sequences shown in Table S3) used in this study were purchased from Eurofins MWG Operon, USA with HPLC/PAGE purification. Nucleic acids were dissolved in double-distilled water before use.

### Yeast Libraries Screening and Colony Sequencing

Screens were performed using a yeast-based strategy based on mouse and zebrafish libraries containing random genomic fragments cloned upstream of the yeast *URA3* gene [13]. A total of 2×10^8^ yeast cells of the haploid strain *MATa* BY404 containing zebrafish or mouse genomic DNA were separately mated to an equal number of the haploid strain *MATα* (W303) containing a pYoh-1 plasmid carrying the zebrafish *cnbp* cDNA coding sequence fused to the GAL4 activating domain. Yeast growth, mating and selection were performed as described elsewhere [13]. Mating efficiency was calculated by the number of diploid colonies on plates selecting for diploid yeast on Trp^-^ Ade^-^ plates per total colony number on YPD plates. Diploid yeast were grown in Ura^-^ medium at 30°C to select for Ura+ (*URA3*) phenotype, clones were single-colony picked and checked for diploidy (Trp^+^ Ade^+^) and re-checked for the Ura^+^ phenotype. For colony-PCR, plated yeast colonies were lysed in 0.02N NaOH for 10 minutes at 95°C. Alternatively, yeast plasmids were purified using the ZymoprepTM Yeast Plasmid Miniprep II kit (ZYMO RESEARCH, Orange, CA, USA) according to the manufacturer’s instructions. First round of PCR reactions was performed using Forward366 and ReverseUra oligonucleotides (Table S3). PCR conditions were: 2.5 mM MgCl_2_, 2 mM of each oligonucleotide, 0.2 mM dNTP and 1.25 units of Taq (Invitrogen) per reaction in a final volume of 20 µl. Thermal cycling was: 95°C for 5 minutes, 40 cycles of 30 s 95°C, 30 s 55°C, 1 min 72°C, and a final extension step of 72°C for 5 minutes. When necessary, nested PCR was performed using Forward366 and Reverse366 and one microliter of a 1∶10^5^ dilution of each Forward366/ReverseUra PCR reactions. Amplification conditions were: 2.0 mM MgCl_2_, 0.4 mM of each oligonucleotide, 0.2 mM dNTP and 1.25 units of Taq (Invitrogen) per reaction in a final volume of 20 µl. Thermal cycling was 95°C for 5 minutes, 35 cycles of 30 s 95°C, 30 s 63°C, 30 s 72°C, and a final extension step of 72°C for 5 minutes. PCR products were sequenced using Reverse366 and/or Forward366 oligonucleotides by Macrogen Corp. or at the Maine University Sequencing Facilities.

### Sequence Analysis

Sequences were submitted to BLAST (http://blast.ncbi.nlm.nih.gov/) and analyzed with the CLC sequence viewer software (http://www.clcbio.com). DNA sequences were mapped to the mouse or zebrafish genomes using BLAST/BLAT genome browsers (ENSEMBL; http://www.ensembl.org/Multi/blastview or UCSC Genome Browser: http://genome.ucsc.edu/cgi-bin/hgBlat?command=start). Promoter regions were defined as <1 kbp from reported transcription start sites and intergenic regions were defined as regions spanning <10 (not including promoter region) or >10 kbp from both ends of reported transcriptional units. Genes surrounding the mapped sites were searched from the ENSEMBL or UCSC genome browsers, listed, and their relative positions registered. Genomic and gene sequences were downloaded using Ensembl Biomart (http://www.ensembl.org/biomart/martview) and genome versions ZV9 for zebrafish and NCBIM37 (mm9) for mouse genomes.

### Searching and Mapping the CNBP DNA Consensus Binding-site

Motif discovery within sequences were performed using the Consensus [37], MEME [38] and Gibbs Motif Sampler [39] algorithms implementing the Tmod software [14]. Default settings were used for each algorithm with the exception that different motif lengths were scanned. The obtained consensuses were graphed using WebLogo (http://weblogo.berkeley.edu/). Putative CNBP binding sites were mapped in selected sequences using MEME/MAST (Motif Alignment and Search Tool) at the MEME website (http://meme.nbcr.net/meme/intro.html). Parameters were set as default and only those sequences with E-value <1e-^04^ were kept for further studies.

### Guanine-quadruplex Structure Search

Putative Quadruplex forming Sequences (PQS) were searched using Quadparser software (http://www.quadruplex.org/?view=quadparser). Parameters were set to search for quadruplexes formed by the stacking of two (G2) or three (G3) guanine quartets with loop sequence lengths of 1 to 15 nucleotides. PQS density was calculated as the ratio of the number of PQS in each sequence (or genome) per kbp analyzed, as in [40].

### Bioinformatic Gene Data Analysis

Gene Ontology (GO) data and GO term enrichment was analyzed with BiNGO plugin using Cytoscape v2.8 (http://www.cytoscape.org/). Genes of the synexpression group were searched in *Danio rerio* and *Mus musculus* using a microarray based co-expression software COXPRESdB (http://coxpresdb.jp/) and *in situ* hybridization data from Zebrafish Model Organism (ZFIN) (http://zfin.org/) and EMAGE gene expression (http://www.emouseatlas.org/emage/) databases. Gene comparisons were made using BioVenn (http://www.cmbi.ru.nl/cdd/biovenn/).

### Microinjection of Antisense Morpholinos

Embryos were obtained by natural mating and injected with spl-MO and mis-MO as described elsewhere [5]. Briefly, morpholinos were designed and synthesized by Gene Tools (Philomath, OR, USA) with sequences spl-MO: 5′-TATCTCTTCTTAGCTTACCCTTTCC-3′, mis-MO: 5′-ATgCAAAAgACTgACTGcTACTgAT-3′ (lower cases indicate the five mispaired bases). Embryos were injected with 5 nl of 1.75 µg/µl morpholino oligonucleotide solution prepared in Danieau 1X at the one-cell stage into the yolk immediately below the blastodics using a gas-driven microinjection apparatus (MPPI-2 Pressure Injector, Applied scientific Instrumentation; Eugene, OR, USA). Microinjection experiments were independently repeated three times using different embryo lays. Morphant and control embryos were used for gene expression quantitation by qRT-PCR and whole-mount *in situ* hybridization as detailed below.

### Gene Expression Quantitation by Real-time Quantitative Reverse Transcription PCR (qRT-PCR)

Total RNA from 24-hpf embryos was obtained using TRIZOL Reagent (Invitrogen) following the manufacturer’s instructions. Purified RNA was incubated with RQ1 DNAse (Promega) and oligo dT retro-transcribed with MMLV reverse transcriptase (Promega). Quantification reactions were performed using three different RNA purifications from three independent microinjection experiments using an Eppendorf Realplex2 apparatus and SYBR green I (Invitrogen) chemistry. Briefly, each reaction tube (20 µl) consisted of 0.5× SYBR green I, 0.1 µM of forward primer, 0.1 µM of reverse primer, 2.5 mM MgCl_2_, 0.2 mM dNTPs, 0.5 U Platinum Taq DNA polymerase (Invitrogen) and 2 µl of template/negative controls. Templates were 1∶20 diluted cDNA samples. After an initial denaturation step (94°C for five minutes), 40 amplification cycles were performed, with each cycle consisting of 94°C for 20 s, 30 s at 63°C and an extension step of 30 s at 68°C. *Ef1α* and *rpl13α* were used as endogenous control for gene normalization [41]. Primer sequences for *p53, p21, tbx2b* and *cnbp* were as reported elsewhere [33]. Specific oligonucleotide primers for putative CNBP targets and neural crest marker genes were designed using Primer-BLAST (http://www.ncbi.nlm.nih.gov/tools/primer-blast/) and their specificity checked using MFE primer 2.0 (http://biocompute.bmi.ac.cn/CZlab/MFEprimer-2.0/). Primer sequences used are shown in Table S3. Relative gene expression values were calculated as described elsewhere [33]. P-values were obtained from *t*-student analysis (P<0.05 was considered as significant).

### Electrophoretic Mobility Shift Assays (EMSAs)

EMSAs were performed as described previously [15]. Briefly, binding reactions were carried out in a final reaction volume of 20 µl for 30 min at 37°C. ^32^P-labeled single-stranded probes (Table S3) were added to a final concentration of 2 nM in the presence of increasing amounts (specified in figure legends) of purified recombinant zCNBP. Reactions were subjected to electrophoresis on 12% (for 14-nucleotide probes) or 10% (for 30-nucleotide probes) polyacrylamide gels containing 5% glycerol in 0.5× TBE buffer. Gels were heat-vaccum dried, exposed to a Storage Phosphor Screen (Amersham Biosciences/GE Healthcare), and subsequently scanned on a STORM 860 PhosphorImager using ImageQuant 5.2 software. Apparent dissociation constants (*K*_d_) were estimated from the intensity of radioactive bands, as described previously [16].

### Expression and Purification of Recombinant Zebrafish CNBP (zCNBP)

Recombinant zCNBP was obtained by overexpressing in *E. coli* a chimeric thioredoxin(Trx)-His_6_-TEV-zCNBP protein. zCNBP chimera was affinity purified using a Ni^2+^ column, subsequently treated with TEV protease, and then subjected to a second round of Ni^2+^ affinity chromatography. zCNBP purity was checked by SDS-PAGE, and protein concentration measured as Lowry et al. [42].

### Chromatin Immuno Precipitation (ChIP)

Zebrafish embryos were obtained by natural mating and injected at the one-cell stage with a capped-mRNA coding for the wild type CNBP fused to the eGFP, as described in [4]. Fluorescent 24-hpf embryos were selected and used for ChIP experiments. Briefly, 100 24-hpf embryos were disrupted and crosslinked as described in [43]. Cellular pellets were resuspended in 200 µl of SDS Lysis buffer (1% SDS, 10 mM EDTA, 50 mM Tris-HCl pH 8.1, plus protease inhibitors cocktail). Chromatin was sheared to an average size of 0.3 to 0.5 kbp using Bioruptor Sonicator (Diagenode) set up at 30 seconds on/30 seconds off, high intensity, for a total sonication time of 7.5 min. Chromatin immunoprecipitations were performed using a Chromatin Immunoprecipitation Assay Kit (Millipore) and anti-eGFP ChIP-grade antibodies (Ab290 Abcam). Antibody, no-antibody, and Input samples were processed according to the manufactureŕs instructions, resuspended in 50 µl of milliQ water, and used for PCR assays. Each reaction tube (20 µl) consisted of 0.2 µM of forward primer, 0.2 µM of reverse primer, 2.5 mM MgCl_2_, 0.2 mM dNTPs, 0.5 U Taq DNA polymerase (Invitrogen) and 1 µl of each sample. In the case of *tbx2b* (*t*2), 5% DMSO was added to reaction medium, while *wnt5b* (*w*1) and *wnt5b* (*w*2) amplification required HOTFIREpol polymerase plus 1X S solution (Solis Biodine). Cycling conditions were 94°C for 10 min, 45 amplification cycles (each cycle consisting of 30 s at 94°C, 30 s at 63°C and 30 s at 68°C), and a final extension step of 7 min at 68°C. Primer sequences are shown in Table S3. PCR products were run in 2% agarose gel and stained with Gel Green.

### Whole-mount *in situ* Hybridization (ISH)

Control and morphant embryos staged at 15- and 24- hpf were fixed overnight in 4% (w/v) paraformaldehyde (PFA) in phosphate-buffered saline (PBS) at 4°C. After washing, embryos were stored in methanol at −20°C until their use. Whole-mount *in situ* hybridizations was carried out using pools of embryos obtained from three independent microinjection experiments following previously described procedures [44]. Digoxigenin-UTP-labeled riboprobes were synthesized by *in vitro* transcription using linearized cDNA cloned in plasmids for *tbx2b* [45] and *wnt5b* [46]. *Smarca5* cDNA was obtained by RT-PCR on 6- to 72-hpf staged embryos total RNA using primers shown in Table S3, and then cloned in pGEM-T Easy vector (Promega). *Cnbp* probe was prepared as previously reported [47]. After microscopic registration, embryos were re-fixed in 4% (w/v) PFA in PBS at 4°C during 24 hours. Finally, embryos were observed with a MVX10 Olympus Microscope and a recorded with a MVXTV1XC Olympus digital camera.

### *Cnbp* Gene Network Construction

The proposed gene network was constructed using Biotapestry v5.0.2 (http://www.biotapestry.org/).

## Supporting Information

Figure S1 **EMSAs of control and mutant CNBP-binding sites. (A)** EMSAs were performed using labeled ssDNA control probes containing the 14-nucleotide consensus G-rich consensus sequence plus 8-nucleotide A/T rich flanking sequences (C30), a mutant probe in which the six more conserved guanines of the consensus were replaced by adenine (C30- mut), a C-rich probe complementary to the consensus sequence (C30-comp), and a doublestranded probe (ds-C30). Only C30 is bound by CNBP. C30 shows a band of low electrophoretic mobility near the shift position due to the formation of intermolecular secondary structures (probably G-cuadruplex since they are reduced using lithium as the main cation in the solution). **(B)** EMSAs were performed using labeled mutant versions of ssDNA probes containing the predicted consensus-like CNBP-binding sites from candidate targets. CNBP binding to mutant probes is clearly reduced except for *t2* and *w2*. This may be due to the G-rich sequences surrounding the consensus-like CNBP-binding sites in these probes (see Table S3). In all cases, increasing concentrations of recombinant zebrafish CNBP (0.015, 0.05, 0.15, 0.5, 1.5 and 5 µM) were used. Arrows at the left of the gels indicate free and shifted probes.
(TIF)

Figure S2 **Western Blot of CNBP-eGFP transiently expressed in zebrafish embryos.** CNBP-eGFP fusion protein was detected using specific anti-eGFP antibody in injected (+) or control 24 hpf-embryo protein extracts (−). Zebrafish embryos were processed for western blotting according to Weiner, et al. (2007)^4^. Briefly, 24-hpf embryos were homogenized and a proportion of the extracts corresponding to five embryos per well were loaded onto a 12% acrylamide gel for SDS–PAGE. Proteins were electro-transferred to Nitrocellulose membrane and blocked overnight with TBS supplemented with 5% (w/v) of milk and 0.1% of Tween 20. After washing, the membrane was incubated for 2 h with a rabbit antibody against eGFP (Abcam, Ab290) diluted 1/5,000 in TBS and then for 1 h with an anti-rabbit Ig HRP-linked antibody (Amersham Life Biosciences) diluted 1/5,000. The reaction was developed with ECLTM Western Blotting Analysis System (Amersham Biosciences, UK) using Ortho CP-G Plus X-ray films (Agfa-Gevaert, Argentina). ^4^ Weiner AM, Allende ML, Becker TS, Calcaterra NB (2007) CNBP mediates neural crest cell expansion by controlling cell proliferation and cell survival during rostral head development. *J. Cell Biochem.*, **102,** 1553–1570.
(TIF)

Table S1 **Clone Data and Analysis.**
(XLSX)

Table S2 **Consensus Sequence Data Analysis.**
(XLSX)

Table S3 **Sequences of oligonucleotides and primers used in this study.**
(PDF)

Table S4 **Gene lists and Gene Ontology Data.**
(XLSX)

Table S5 **COXPRESdB Data for CNBP.**
(XLSX)

Table S6 **Synexpression Data for CNBP.**
(XLSX)
