# Supplementary figures and images for: Beyond the Binding Site: In Vivo Identification of tbx2, smarca5 and wnt5b as Molecular Targets of CNBP during Embryonic Development
Source: PLoS One. 2013 May 7;8(5):e63234. doi: 10.1371/journal.pone.0063234 (PMC3646763; doi:10.1371/journal.pone.0063234)

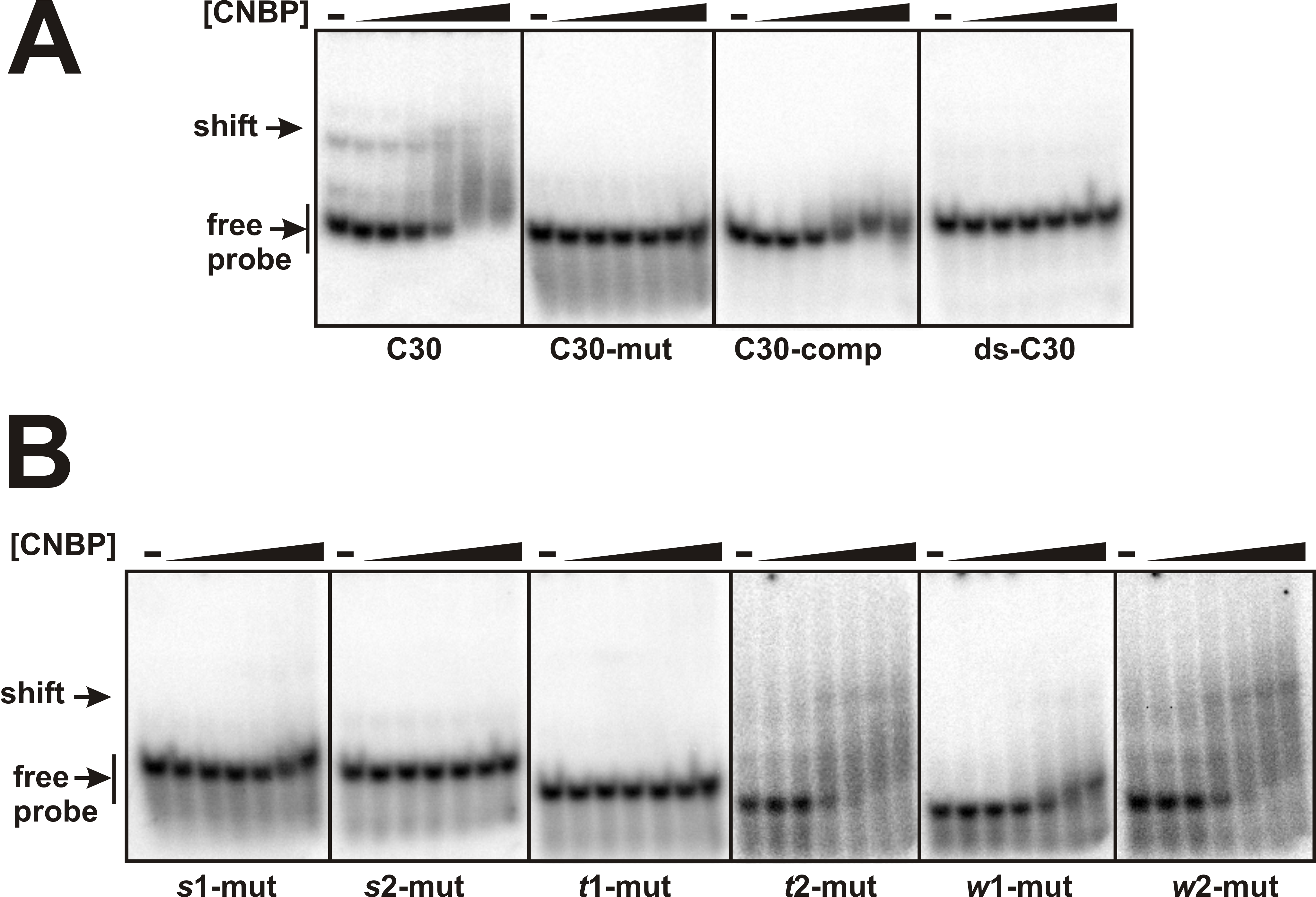

Supplement: Figure S1 — EMSAs of control and mutant CNBP-binding sites. (A) EMSAs were performed using labeled ssDNA control probes containing the 14-nucleotide consensus G-rich consensus sequence plus 8-nucleotide A/T rich flanking sequences (C30), a mutant probe in which the six more conserved guanines of the consensus were replaced by adenine (C30- mut), a C-rich probe complementary to the consensus sequence (C30-comp), and a doublestranded probe (ds-C30). Only C30 is bound by CNBP. C30 shows a band of low electrophoretic mobility near the shift position due to the formation of intermolecular secondary structures (probably G-cuadruplex since they are reduced using lithium as the main cation in the solution). (B) EMSAs were performed using labeled mutant versions of ssDNA probes containing the predicted consensus-like CNBP-binding sites from candidate targets. CNBP binding to mutant probes is clearly reduced except for t2 and w2. This may be due to the G-rich sequences surrounding the consensus-like CNBP-binding sites in these probes (see Table S3). In all cases, increasing concentrations of recombinant zebrafish CNBP (0.015, 0.05, 0.15, 0.5, 1.5 and 5 µM) were used. Arrows at the left of the gels indicate free and shifted probes. (TIF) [file pone.0063234.s001.tif]

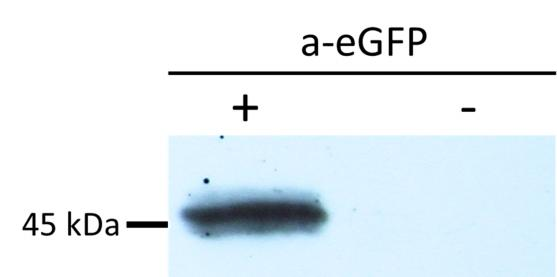

Supplement: Figure S2 — Western Blot of CNBP-eGFP transiently expressed in zebrafish embryos. CNBP-eGFP fusion protein was detected using specific anti-eGFP antibody in injected (+) or control 24 hpf-embryo protein extracts (−). Zebrafish embryos were processed for western blotting according to Weiner, et al. (2007)4. Briefly, 24-hpf embryos were homogenized and a proportion of the extracts corresponding to five embryos per well were loaded onto a 12% acrylamide gel for SDS–PAGE. Proteins were electro-transferred to Nitrocellulose membrane and blocked overnight with TBS supplemented with 5% (w/v) of milk and 0.1% of Tween 20. After washing, the membrane was incubated for 2 h with a rabbit antibody against eGFP (Abcam, Ab290) diluted 1/5,000 in TBS and then for 1 h with an anti-rabbit Ig HRP-linked antibody (Amersham Life Biosciences) diluted 1/5,000. The reaction was developed with ECLTM Western Blotting Analysis System (Amersham Biosciences, UK) using Ortho CP-G Plus X-ray films (Agfa-Gevaert, Argentina). 4 Weiner AM, Allende ML, Becker TS, Calcaterra NB (2007) CNBP mediates neural crest cell expansion by controlling cell proliferation and cell survival during rostral head development. J. Cell Biochem., 102, 1553–1570. (TIF) [file pone.0063234.s002.tif]
