# Supplementary material for: Beyond the Binding Site: In Vivo Identification of tbx2, smarca5 and wnt5b as Molecular Targets of CNBP during Embryonic Development
Source: PLoS One. 2013 May 7;8(5):e63234. doi: 10.1371/journal.pone.0063234 (PMC3646763; doi:10.1371/journal.pone.0063234)
Supplement: Table S3 — Sequences of oligonucleotides and primers used in this study. (PDF) [file pone.0063234.s005.pdf]

Supplementary Table 3: Sequences of oligonucleotides and primers used in this study

Experiment	Name	Length (bases)	Sequence
EMSA	C14	14	5′-**GAGGGGGAGGGGGG**-3′
	C14-mut	14	5′-**GAAGAAGAAGAGAG**-3′
	C14-comp	14	5′-**CCCCCCTCCCCCTC**-3′
	C30	30	5′-TAATAATA**GAGGGGGAGGGGGG**ATAATAAT-3′
	C30-mut	30	5′-TAATAATA**GAAGAAGAAGAGAG**ATAATAAT-3′
	C30-comp	30	5′-ATTATTAT**CCCCCCTCCCCCTC**TATTATTA-3′
	*s*1	30	5′-GCACCCTG**GAGGAGGAGGAAGG**GTTGGTAG-3′
	*s*1-mut	30	5′-GCACCCTG**GAAGAAGAAGAAAG**GTTGGTAG-3′
	*s*2	30	5′-GAAGCTCA**GAGAGGTAAAGGGG**TGCAGCAC-3′
	*s*2-mut	30	5′-GAAGCTCA**GAAAAATAAAAGAG**TGCAGCAC-3′
	*t*1	30	5′-GATGGAGA**GAGTGGGAGAGAAG**AAGCAGAG-3′
	*t*1-mut	30	5′-GATGGAGA**GAATAAGAAAAAAG**AAGCAGAG-3′
	*t*2	30	5′-AAAAAGGG**GAGGGGGGGGGGGG**GGGGGGGT-3′
	*t*2-mut	30	5′-AAAAAGGG**GAAGAAGGAGAGAG**GGGGGGGT-3′
	*w*1	30	5′-CACACCAG**GTATGGGGGGGGGG**GAATGGGT-3′
	*w*1-mut	30	5′-CACACCAG**GTATAAGGAGAGAG**GAATGGGT-3′
	*w*2	30	5′-AGTGTGGT**GGGTGGGAGGGGGG**TTGGGGGG-3′
	*w*2-mut	30	5′-AGTGTGGT**GGATAAGAAGAGAG**TTGGGGGG-3′
**Bold** indicates the 14-nucleotide consensus or consensus-like sequences.<br>Grey shade indicates mutations in the 14-nucleotide consensus or consensus-like sequences.<br>Underline indicates variations in the consensus-like sequences found in genomic sequences in respect to the 14-nucleotide consensus sequence.			
PCR	*Yeast Colony PCR*		
	Forward366	27	5´-GCGCTTTAAGAGAAAATATTTGTCCTG-3´
	ReverseUra	26	5´-GTAGCAGCACGTTCCTTATATGTAGC-3´
	Reverse366	32	5´-CGGCTATTTCTCAATATACTCCTAATTAATAC-3´
	qRT-PCR		
	asphf	25	5´-CAAGGACATGTTTATGCTGGCAGGT-3´
	asphr	21	5´-GCGCATGCACGCTCATTCACC-3´
	col10a1f	25	5´-CCTACAGCATGCATGTGAATGGAGC-3´
	col10a1r	25	5´-CCGGACATCTGGTCCACAAATCCCT-3´
	smarca5f	23	5´-TCAAGTCTCGCACAGCCATGGAG-3´
	smarca5r	22	5´-TTCTGAGCCGAAGATGTGCGCG-3´
	wnt5bf	22	5´-AGCCGGAAGAATGGCGGTGTAT-3´
	wnt5br	20	5´-GCACGAGCCTGAGACGCCAT-3´
	ap2af	22	5´-CCCTCAAAGCACCACAAGAGCA-3´
	ap2ar	21	5´-GGCATGGGAGCTATTGCCAGC-3´
	sox9bf	20	5´-GGACATCGGCGAGCTGAGCA-3´
	sox9br	20	5´-CTGGAGAACCCTGCACCGGC-3´
	sox10f	24	5´-GCGCACCTTTATTTCACACAAGCA-3´
	sox10r	21	5´-TGGACTTGAGGCACTAGCGGT-3´
	ISH probe		
	ish-smarca5f	21	5´-GCCAACGAGAAGTGGGGACGA-3´
	ish-smarca5r	21	5´-TCAGGGGTCCCGTCCTGCTTG-3´
	ChIP		
	s1f	20	5´-AGACTTACCGATTGCCCCTG-3´
	s1r	22	5´-GCAACAGCATGGATACAGCTTC-3´
	t1f	20	5´-AGGGTCGGGTTACGGTTTTG-3´
	t1r	20	5´-CGAATCACCCAACCGAGTCA-3´
	t2f	20	5´-TTGTCTTACAGCGGACACCG-3´
	t2r	20	5´-GCACTGCCACTGTTTACTGC-3´
	w1f	21	5´-TGACTGAACTGGCTGTCTCAC-3´
	w1r	22	5´-ACCAGTGTGTAGCATCCAGTTG-3´
	w2f	22	5´-CAACTGGATGCTACACACTGGT-3´
	w2r	24	5´-TCAATCAGATCAGGTGAAAACAGTG-3´
